# Management of Abdominal Aortic Aneurysm Surgery in Glanzmann’s Thrombasthenia Patients with Anti-GPIIb-IIIa Antibodies: A Case Report

**DOI:** 10.3390/jcm13195839

**Published:** 2024-09-30

**Authors:** Alexandre Leuci, Antoine Millon, Alice Chopin, Hamdi Rezigue, Ssakher Alotaibi, Yesim Dargaud

**Affiliations:** 1 UR4609 Hemostasis & Thrombosis Research Unit, Faculté de Médecine Lyon Est, Université Claude Bernard Lyon 1, 69008 Lyon, France; alexandre.leuci@univ-lyon1.fr; 2 Service de Chirurgie Vasculaire, Hopital Louis Pradel, Hospices Civils de Lyon, 69500 Lyon, France; antoine.millon@chu-lyon.fr; 3 Service d’Anesthésie Réanimation, Hopital Louis Pradel, Hospices Civils de Lyon, 69500 Lyon, France; alice.chopin@chu-lyon.fr; 4 Centre de Biologie Est, Laboratoire d’Hematologie, Hopital Louis Pradel, Hospices Civils de Lyon, 69500 Lyon, France; hamdi.rezigue@chu-lyon.fr; 5 Unite d’Hemostase Clinique, Hopital Louis Pradel, Hospices Civils de Lyon, 69500 Lyon, France; sshaker.alotaibi@chu-lyon.fr

**Keywords:** Glanzmann thrombasthenia, anti-GPIIb-IIIa antibodies, recombinant factor VIIa, aortic aneurysm, antiplatelet agents

## Abstract

Glanzmann’s thrombasthenia (GT) is a rare autosomal recessive disorder of platelet function. The frequent occurrence of alloimmunization due to repeated platelet transfusions is the major complication of the disease. Achieving hemostasis in these patients with anti-GPIIb-IIIa antibodies during surgical procedures is a significant challenge due to the high risk of bleeding. Recombinant activated factor VII (rFVIIa) is an effective agent for achieving hemostasis in alloimmunized Glanzmann’s thrombasthenia patients. The key clinical question was to determine whether abdominal aortic aneurysm surgery can be safely performed with rFVIIa in Glanzmann’s thrombasthenia patients with anti-GPIIb/IIIa antibodies and whether long-term antiplatelet therapy is suitable for these patients. The patient underwent endovascular aneurysm repair with intensive rFVIIa administration, experiencing neither bleeding nor thrombosis. Data regarding the surgical management of Glanzmann’s thrombasthenia patients with anti-GPIIb-IIIa antibodies and the use of antithrombotics in this high-risk population are still very limited. Sharing clinical experience can be valuable for hematologists managing similar cases.

## 1. Introduction

Glanzmann’s thrombasthenia is a rare autosomal recessive disorder characterized by spontaneous mucocutaneous bleeding that varies in frequency and severity, even among family members, and can occasionally be life-threatening [1]. Excessive bleeding from trauma or surgery is also common [2]. In Glanzmann’s thrombasthenia, thrombus formation fails due to a deficiency or dysfunction of the αIIbβ3 integrin (GPIIb-IIIa), a critical platelet receptor. This integrin mediates the final step of platelet aggregation by binding fibrinogen and other adhesive proteins, thereby facilitating platelet cross-linking [3]. Upon platelet activation, αIIbβ3 changes conformation to expose binding sites for these proteins. It also transmits forces for clot contraction and transports fibrinogen to α-granules for storage. Mutations in Glanzmann’s thrombasthenia differentially affect the expression and function of αIIbβ3, resulting in varying degrees of bleeding severity [3]. For invasive procedures, platelet transfusion in association with tranexamic acid is the standard of care for patients with Glanzmann’s thrombasthenia [4]. However, approximately 20–30% of patients may develop an immune response against the deficient αIIbβ3 complex or HLA class I system, rendering transfusions ineffective [5]. In addition, animal models suggest that the hemostatic efficacy of transfused platelets is reduced in the absence of thrombocytopenia, as dysfunctional platelets in Glanzmann’s thrombasthenia may interfere with the activity of transfused platelets. For patients who are refractory to platelet transfusions, recombinant factor VIIa (rFVIIa) is the first-line alternative hemostatic agent [4]. High-dose rFVIIa supports hemostasis primarily through a TF-independent mechanism by binding to the negatively charged phospholipid surface on activated platelets and improving platelet adhesion and aggregation via the GPIb/IX/V complex. It also activates FX to FXa, resulting in a thrombin burst that improves fibrin clot structure, reduces clot permeability, and tightens the fibrin network, thereby improving hemostasis [4,5]. Surgical procedures in patients with Glanzmann’s thrombasthenia are challenging due to the high risk of bleeding. Although several articles have documented major surgical procedures in Glanzmann’s thrombasthenia patients [6,7,8,9], experience in patients with neutralizing anti-GPIIb-IIIa antibodies (inhibitors) is limited [10]. To our knowledge, no major vascular surgery has been reported in patients with Glanzmann’s thrombasthenia who are refractory to platelet transfusion.

We report the surgical and hemodynamic strategies used in a patient with Glanzmann’s thrombasthenia and anti-GPIIb-IIIa antibodies who successfully underwent elective endovascular repair of an aortic aneurysm. The challenge was two-fold: (i) effectively managing the risk of bleeding in a patient with a high bleeding risk who is refractory to platelet transfusions during major vascular surgery, and (ii) prescribing long-term antithrombotic therapy for a patient with Glanzmann thrombasthenia and inhibitors.

## 2. Case Report

**Figure 1:**
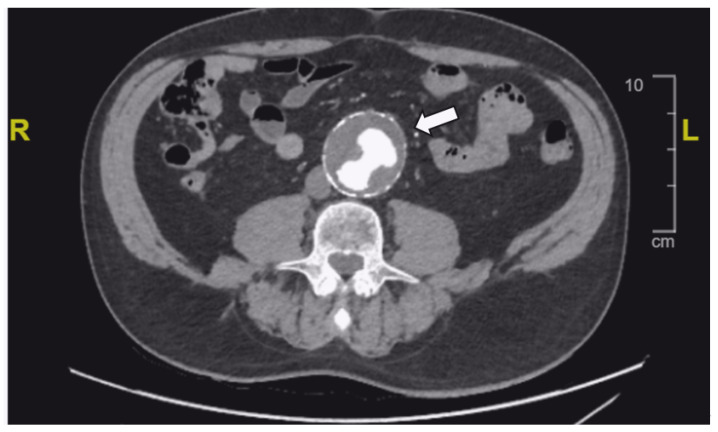
The CT scan demonstrating the large abdominal aortic aneurysm (arrow).

A 75-year-old man carrying the Gypsy mutation, which modifies the splice donor site of intron 15 of the GPIIb gene and is responsible for hereditary type I Glanzmann thrombasthenia, with anti-GPIIb-IIIa alloimmunization, experienced multiple trauma- and surgery-induced bleeding episodes. In 1978, he underwent uneventful surgery to remove a metallic foreign body from his neck with platelet concentrate substitution. In 1999, surgery for vocal cord nodules, supported by platelet transfusions, was complicated by severe bleeding, with the monoclonal antibody immobilization of platelet antigens (MAIPA) test revealing anti-GPIIb-IIIa antibodies. In the same year, an admission to the gastroenterology department for melena led to a colonoscopy, during which three polyps were removed with rFVIIa 90 μg/kg q.2h. In 2007, a tonsillar hemorrhage required intensive care at the University Hospital of Brussels. Despite treatment with rFVIIa, persistent bleeding necessitated angiography, which revealed a lingual artery saccular aneurysm requiring embolization. This was followed by injections of rFVIIa and tranexamic acid. In 2009, a Mallory Weiss lesion was diagnosed during a gastrointestinal bleeding episode related to chronic alcoholism. In 2014, an emergency hospitalization for meningeal hemorrhage and multiple facial wounds after an alcohol-induced fall was treated with rFVIIa. The patient also underwent multiple dental extractions and skin tumor excisions under successful rFVIIa treatment. In 2023, an large abdominal aortic aneurysm requiring surgical treatment was discovered. The CT scan revealed a complex juxtarenal abdominal aortic aneurysm with a maximum diameter of 57 mm, lacking a healthy proximal landing zone below the renal arteries but with adequate distal iliac landing zones. The celiac trunk, superior mesenteric artery, and both renal arteries were free of stenosis and suitable for a custom-made four-fenestration stent graft (Figure 1).

**Figure 2:**
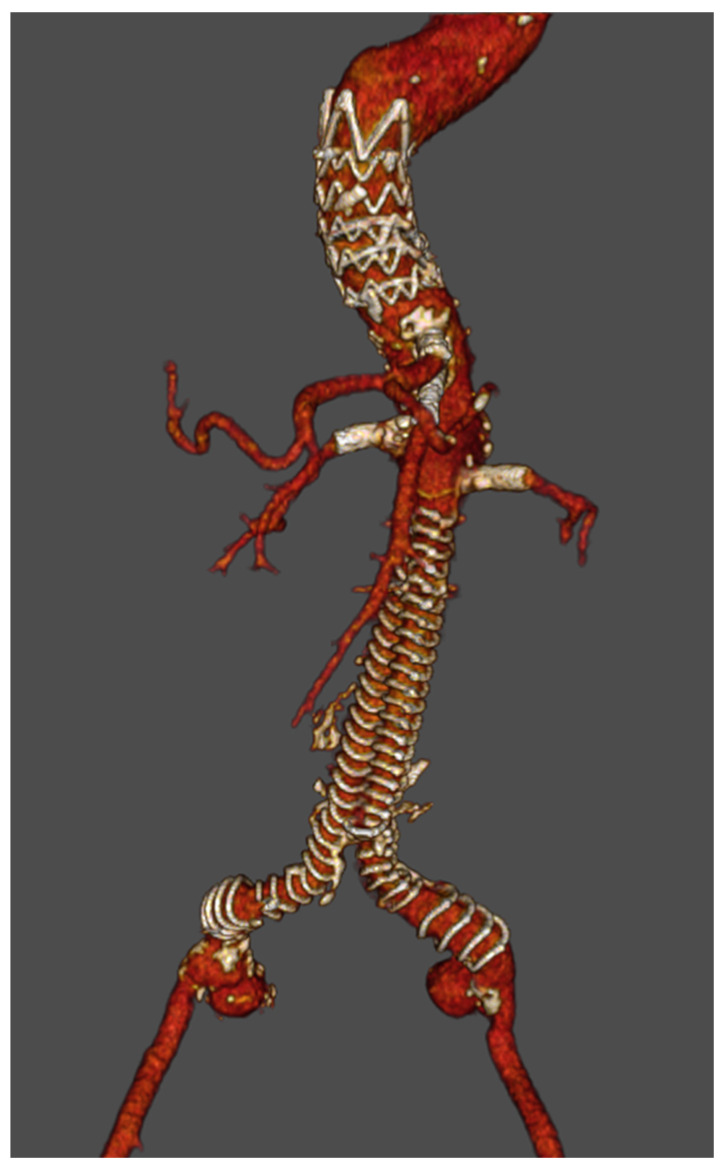
A post-operative CT scan 3D reconstruction showing an exclusion of the abdominal aortic aneurysm.

A multidisciplinary team consisting of specialists in hematology, vascular surgery, anesthesia, and laboratory medicine was assembled to optimize perioperative management. The patient received rFVIIa (NovoSeven, Novonordisk, Bagsvaerd, Denmark) at a dose of 90 µg/kg, initiated 15 min before surgery and then administered every 2 h for the first 24 h. On post-operative days one and two, rFVIIa was continued at the same dose with administrations every 4 and 8 h, respectively. The patient presented with a juxtarenal abdominal aortic aneurysm, for which an endovascular repair strategy using a custom-made 4-fenestrated stent graft was chosen. The procedure was successfully performed under general anesthesia. A full dose of heparin (8500 IU) was administered to achieve a target activated clotting time (ACT) between 200 and 250. All targeted vessels (including the celiac trunk, superior mesenteric artery, and both left and right renal arteries) were stented successfully without any complications. Post-operative CT confirmed exclusion of the abdominal aortic aneurysm (AAA) with good patency of both the stent graft and the bridging stent (Figure 2).

Total operative time was 135 min with minimal blood loss of less than 250 mL. There was no surgery-related bleeding complication and no red blood cell transfusion was required during the procedure. Heparin reversal with protamine was performed after hemostasis was achieved, resulting in normalization of the activated clotting time (ACT). Fortunately, no life-threatening complications occurred, and the patient was subsequently transferred to the intensive care unit (ICU) for further monitoring. Pre-operative hemoglobin levels were recorded at 10 g/dL and 9.6 g/dL on post-operative day 1. An antithrombotic treatment with low-dose aspirin (75 mg/day) was prescribed post-operatively and was discontinued three months later due to frequent gingival bleeding.

## 3. Discussion

The management of patients with Glanzmann’s thrombasthenia and anti-GPIIb-IIIa antibodies during major surgery remains challenging, despite the availability of clinical practice guidelines for the optimal management of invasive procedures and surgical bleeding [11,12]. Cardiovascular surgery in particular poses additional challenges due to the need for long-term post-operative antithrombotic treatment, which can be complicated in this patient population due to their increased risk of major bleeding [13]. Few studies have reported on such complex scenarios involving the use of dual antiplatelet therapy [14] or anticoagulants in Glanzmann’s thrombasthenia patients without anti-GPIIb-IIIa antibodies [6,15,16]. Major cardiovascular surgery can be performed in Glanzmann’s thrombasthenia patients without anti-GPIIb-IIIa antibodies, but it requires a tailored approach that carefully considers bleeding tendencies, clinical history, and thromboembolic risk when prescribing post-operative antithrombotic agents [17,18]. To our knowledge, no publication has documented the use of antithrombotic therapy in patients with Glanzmann thrombasthenia and anti-GPIIb-IIIa antibodies. Traditionally, dual antiplatelet therapy has been the gold standard for preventing stent thrombosis for at least 3 months following percutaneous coronary intervention [19]. However, data on antiplatelet therapy after peripheral stenting are limited, and the decision between dual and monotherapy varies significantly across surgical practices [20]. Glanzmann’s thrombasthenia, characterized by deficient or dysfunctional glycoprotein IIb-IIIa, leads to a bleeding diathesis. While antiplatelet therapy traditionally targets this platelet membrane protein. In our case, which involved a patient with anti-GPIIb-IIIa antibodies, we decided to initiate indefinite low-dose aspirin therapy to prevent thrombotic risks associated with endoprostheses and vascular stents. However, the patient experienced recurrent gingival bleeding, which resulted in anemia. As a result of recurrent daily bleeding, aspirin was discontinued 3 months after surgery. Binder et al. [21] suggested that daily administration of rVIIa at a dose of 90 µg/kg could potentially serve as an effective prophylactic treatment regimen for Glanzmann’s thrombasthenia patients requiring long-term antithrombotic therapy, such as dual antiplatelet therapy for coronary artery disease. In our patient, no thrombotic complications were observed for over a year following the discontinuation of antiplatelet therapy.

Novel therapeutic approaches such as HMB-001, a bispecific antibody designed for prophylactic treatment to prevent bleeding events in Glanzmann’s thrombasthenia, offer promising prospects. Administered subcutaneously, HMB-001 binds to both endogenous FVIIa and the TREM-like transcript 1 receptor (TLT-1) on activated platelets. This results in the accumulation of FVIIa and its targeted delivery to the surface of activated platelets to achieve hemostasis. Such prophylaxis has the potential to allow the prescription of antithrombotic therapy in Glanzmann’s thrombasthenia patients with anti-GPIIb-IIIa antibodies [22].

## 4. Conclusions

In conclusion, patients with Glanzmann’s thrombasthenia remain susceptible to cardiovascular complications. Here, we present a successful endovascular surgery supported by perioperative rFVIIa treatment in a patient with Glanzmann’s thrombasthenia and antiplatelet alloantibodies. Our case also suggests that discontinuing aspirin after the initial 3 months post-operatively could be a safe option for patients with Glanzmann’s thrombasthenia and anti-GPIIb-IIIa antibodies.

## Data Availability

Data available upon request.
